# Impact of COVID-19 pandemic and diabetes on mechanical reperfusion in patients with STEMI: insights from the ISACS STEMI COVID 19 Registry

**DOI:** 10.1186/s12933-020-01196-0

**Published:** 2020-12-18

**Authors:** Giuseppe De Luca, Miha Cercek, Lisette Okkels Jensen, Marija Vavlukis, Lucian Calmac, Tom Johnson, Gerard Roura i Ferrer, Vladimir Ganyukov, Wojtek Wojakowski, Clemens von Birgelen, Francesco Versaci, Jurrien Ten Berg, Mika Laine, Maurits Dirksen, Gianni Casella, Petr Kala, José Luis Díez Gil, Victor Becerra, Ciro De Simone, Xavier Carrill, Alessandra Scoccia, Arpad Lux, Tomas Kovarnik, Periklis Davlouros, Gabriele Gabrielli, Xacobe Flores Rios, Nikola Bakraceski, Sébastien Levesque, Vincenzo Guiducci, Michał Kidawa, Lucia Marinucci, Filippo Zilio, Gennaro Galasso, Enrico Fabris, Maurizio Menichelli, Stephane Manzo, Gianluca Caiazzo, Jose Moreu, Juan Sanchis Forés, Luca Donazzan, Luigi Vignali, Rui Teles, Francisco Bosa Ojeda, Heidi Lehtola, Santiago Camacho-Freiere, Adriaan Kraaijeveld, Ylitalo Antti, Marco Boccalatte, Iñigo Lozano Martínez-Luengas, Bruno Scheller, Dimitrios Alexopoulos, Giuseppe Uccello, Benjamin Faurie, Alejandro Gutierrez Barrios, Bor Wilbert, Giuliana Cortese, Raul Moreno, Guido Parodi, Elvin Kedhi, Monica Verdoia

**Affiliations:** 1 Division of Cardiology, Azienda Ospedaliero-Universitaria Maggiore della Carità, Università del Piemonte Orientale, Novara, Italy; 2 Centre for Intensive Internal Medicine, University Medical Centre, Ljubljana, Slovenia; 3 Division of Cardiology, Odense Universitets Hospital, Odense, Danemark; 4 University Clinic for Cardiology, Medical Faculty, Ss’ Cyril and Methodius University, Skopje, North Macedonia; 5 Clinic Emergency Hospital of Bucharest, Bucharest, Romania; 6 Division of Cardiology, Bristol Heart Institute, University Hospitals Bristol, NHSFT & University of Bristol, Bristol, UK; 7 Interventional Cardiology Unit, Heart Disease Institute, Hospital Universitari de Bellvitge, L’Hospitalet de Llobregat, Spain; 8 Division of Cardiology, State Research Institute for Complex Issues of Cardiovascular Diseases, Kemerovo,, Russia; 9 Division of Cardiology, Medical University of Silezia, Katowice, Poland; 10 Department of Cardiology, Medisch Spectrum Twente, Thoraxcentrum Twente, Enschede, The Netherlands; 11 Division of Cardiology, Ospedale Santa Maria Goretti, Latina, Italy; 12 Division of Cardiology, St Antonius Hospital, Nieuwegein, The Netherlands; 13 Division of Cardiology, Helsinki University Central Hospital, Helsinki, Finland; 14 Division of Cardiology, Northwest Clinic, Alkmaar, The Netherlands; 15 Division of Cardiology, Ospedale Maggiore, Bologna, Italy; 16 University Hospital Brno, Medical Faculty of Masaryk University Brno, Brno, Czech Republic; 17 H. Universitario y Politécnico La Fe, Valencia, Spain; 18 Hospital Clínico Universitario Virgen de la Victoria, Málaga, Spain; 19 Division of Cardiology, Clinica Villa dei Fiori, Acerra, Italy; 20 Hospital Germans Triasi Pujol, Badalona, Spain; 21 Division of Cardiology, Ospedale “Sant’Anna”, Ferrara, Italy; 22 Maastricht University Medical Center, Maastricht, The Netherlands; 23 University Hospital Prague, Prague, Czech Republic; 24 Invasive Cardiology and Congenital Heart Disease, Patras University Hospital, Patras, Greece; 25 Interventional Cardiology Unit, Azienda Ospedaliero Universitaria “Ospedali Riuniti”, Ancona, Italy; 26 Complexo Hospitaliero Universitario La Coruna, La Coruna, Spain; 27 Center for Cardiovascular Diseases, Ohrid, North Macedonia; 28 Center Hospitalier, Universitaire de Poitiers, University Hospital, Poitiers, France; 29 AUSL-IRCCS Reggio Emilia, Reggio Emilia, Italy; 30 Central Hospital of Medical University of Lodz, Łódź, Poland; 31 Division of Cardiology, AziendaOspedaliera “Ospedali Riuniti Marche Nord”, Pesaro, Italy; 32 Ospedale Santa Chiara di Trento, Trento, Italy; 33 Division of Cardiology, Ospedale San Giovanni di Dio e Ruggi d’Aragona, Salerno, Italy; 34 Azienda Ospedaliero – Universitaria Ospedali Riuniti Trieste, Trieste, Italy; 35 Division of Cardiology, Ospedale “F. Spaziani, Frosinone, Italy; 36 Division of Cardiology, CHU Lariboisière, AP-HP, Paris VII University, INSERM UMRS 942, Paris, France; 37 Division of Cardiology, Ospedale “G Moscati”, Aversa, Italy; 38 Division of Cardiology, Complejo Hospitalario de Toledo, Toledo, Spain; 39 Division of Cardiology, Hospital Clinico Universitario de Valencia, Valencia, Spain; 40 Division of Cardiology, Ospedale “S. Maurizio” Bolzano Ospedale “S. Maurizio”,, Bolzano, Italy; 41 Interventional Cardiology Unit, Azienda Ospedaliera Sanitaria, Parma, Italy; 42 Division of Cardiology, Hospital de Santa Cruz, CHLO - Carnaxide, Carnaxide, Portugal; 43 Division of Cardiology, Hospital Universitario de Canarias, Santa Cruz de Tenerife, Spain; 44 Division of Cardiology, Oulu University Hospital, Oulu, Finland; 45 Division of Cardiology, Juan Ramon Jimenez Hospital, Huelva, Spain; 46 Division of Cardiology, UMC Utrecht, Utrecht, The Netherlands; 47 Division of Cardiology, Heart Centre Turku, Turku, Finland; 48 Division of Cardiology, Ospedale Santa Maria delle Grazie, Pozzuoli, Italy; 49 Division of Cardiology, Hospital Cabueñes, Gijon, Spain; 50 Division of Cardiology, Clinical and Experimental Interventional Cardiology, University of Saarland, Saarbrücken, Germany; 51 Division of Cardiology, Attikon University Hospital, Athens, Greece; 52 Division of Cardiology, Ospedale “A. Manzoni” Lecco, Lecco, Italy; 53 Division of Cardiology, Groupe Hospitalier Mutualiste de Grenoble, Grenoble, France; 54 Division of Cardiology, Hospital Puerta del Mar, Cadiz, Spain; 55 Department of Statistical Sciences, University of Padova, Padova, Italy; 56 Azienda Ospedaliero-Universitaria Sassari, Sassari, Italy; 57 Division of Cardiology, Hospital la Paz, Madrid, Spain; 58 Division of Cardiology, St-Jan Hospital, Brugge, Belgium; 59 Division of Cardiology, Ospedale degli Infermi, ASL Biella, Ponderano, Italy

## Abstract

**Background:**

It has been suggested the COVID pandemic may have indirectly affected the treatment and outcome of STEMI patients, by avoidance or significant delays in contacting the emergency system. No data have been reported on the impact of diabetes on treatment and outcome of STEMI patients, that was therefore the aim of the current subanalysis conducted in patients included in the International Study on Acute Coronary Syndromes–ST Elevation Myocardial Infarction (ISACS-STEMI) COVID-19.

**Methods:**

The ISACS-STEMI COVID-19 is a retrospective registry performed in European centers with an annual volume of > 120 primary percutaneous coronary intervention (PCI) and assessed STEMI patients, treated with primary PCI during the same periods of the years 2019 versus 2020 (March and April). Main outcomes are the incidences of primary PCI, delayed treatment, and in-hospital mortality.

**Results:**

A total of 6609 patients underwent primary PCI in 77 centers, located in 18 countries. Diabetes was observed in a total of 1356 patients (20.5%), with similar proportion between 2019 and 2020. During the pandemic, there was a significant reduction in primary PCI as compared to 2019, similar in both patients with (Incidence rate ratio (IRR) 0.79 (95% CI: 0.73–0.85, *p* < 0.0001) and without diabetes (IRR 0.81 (95% CI: 0.78–0.85, *p* < 0.0001) (p int = 0.40). We observed a significant heterogeneity among centers in the population with and without diabetes (*p* < 0.001, respectively). The heterogeneity among centers was not related to the incidence of death due to COVID-19 in both groups of patients. Interaction was observed for Hypertension (p = 0.024) only in absence of diabetes.

Furthermore, the pandemic was independently associated with a significant increase in door-to-balloon and total ischemia times only among patients without diabetes, which may have contributed to the higher mortality, during the pandemic, observed in this group of patients.

**Conclusions:**

The COVID-19 pandemic had a significant impact on the treatment of patients with STEMI, with a similar reduction in primary PCI procedures in both patients with and without diabetes. Hypertension had a significant impact on PCI reduction only among patients without diabetes. We observed a significant increase in ischemia time and door-to-balloon time mainly in absence of diabetes, that contributed to explain the increased mortality observed in this group of patients during the pandemic.

Trial registration number: NCT 04412655.

## Background

The healthcare systems have dramatically been impacted by global pandemic of coronavirus disease 2019 (COVID-19), with so far more than 56 million cases and more than 1.3 million deaths, especially in Europe, Latin America and United States.

During this pandemic period, most of the resources have understandably been focused on the treatment of COVID-19 patients, thus limiting the access to healthcare services for patients with chronic conditions, whilst being required to warrant the treatment of acute diseases, such as ST-segment elevation myocardial infarction (STEMI). Combined with this diversion of resource, lockdown rules, guidance on social distancing and a public fear of coronavirus contagion appear to have impacted on patient willingness to present to hospital, as evidenced by a reduction in percutaneous coronary intervention (PCI) procedures for ACS, including STEMI [2–7]. While the reduction in STEMI patients has been worldwide described, variations in the referral to primary PCI could be expected within different subsets of patients, and also different outcome effects, with more severe prognostic consequences being expected in higher-risk categories, as among subjects with diabetes [8–11]. However, such issue has never been addressed in dedicated studies, and especially with no analysis based on individual patients’ level data. Therefore, the aim of the present study was to assess the additional impact of diabetes on the management and outcomes of STEMI patients undergoing primary PCI during COVID pandemic.

## Methods

### Study Design and population

The International Study on Acute Coronary Syndromes – ST Elevation Myocardial Infarction (ISACS-STEMI) COVID-19 was established in response to the emerging outbreak of COVID-19 to provide a European snapshot and aimed to estimate the true impact of the COVID-19 pandemic on the treatment and outcome of STEMI patients treated by primary angioplasty [12]. It is a retrospective multicenter registry promoted by the Eastern Piedmont University, Novara, Italy, planned to include at least 40 European primary PCI centers, performing more than 120 primary PCI/year (with expected average > 10/month), with the case load of STEMI not expected to be affected by a potential planned reorganization of the STEMI network. The inclusion period was 2 months (from 1st March until 30th April). The data were compared with those retrospectively collected in the same time window (from 1st March until 30th April) of 2019.

#### Inclusion criteria

STEMI treated by primary angioplasty (including mechanical reperfusion for failed thrombolysis).

#### Data collection

Anonymized data were collected through a dedicated CRF. Each center identified a local Principal Investigator. We collected demographic, clinical, procedural data including total ischemia time, door-to-balloon time (DTB), referral to primary PCI facility, COVID positivity, PCI procedural data, and in-hospital mortality. After collection, each participating center submitted the CRF to the coordinating unit (Eastern Piedmont University), in charge of reporting all data onto the central electronic database. Data were finally checked for missing or contradictory entries.

#### Study outcomes

(1) Number of STEMI patients undergoing percutaneous revascularization; (2) Proportion of patients with ischemia time > 12 h; (3) Proportion of patients with a DTB > 30 min, (4) In-Hospital mortality.

#### Statistics

Data were analyzed using SPSS Statistics Software 23.0 (IBM SPSS Inc., Chicago, Illinois) and R software (version 3.6.2) by an independent statistician (GC). Quantitative variables were described using median and interquartile range. Mean and confidence intervals were obtained assuming Poisson distributions for count data. Incidence rate ratio (IRR) was defined as the ratio between count data in 2020 and 2019, over the same population and time period. Poisson regression models (with log link function) were applied to compare the Incidence rate of Primary PCI per million of residents with and without diabetes [13] per-year in 2020 with the same rate in 2019, correcting for possible impact of major risk factors [14]. Details are described in the supplementary appendix. Analysis was also conducted according to major European geographic areas (see supplementary materials) and subgroups of patients such as according to age, gender, and hypertension. Associations of the IRR (on logarithmic scale) with COVID disease and COVID mortality were tested with Poisson models, and a correlation measure was also provided by the Pearson’s index.

A subsequent analysis was based on individual data, who were grouped according to the year of the intervention (2019 vs 2020). Absolute frequencies and percentages were used for qualitative variables. ANOVA or Mann–Whitney and chi-square test were used for continuous and categorical variables, respectively. Normal distribution of continuous variables was tested by the Kolmogorov–Smirnov test).

Multivariable logistic regression analyses were performed to identify the impact of the year of intervention on time delays and mortality after adjustment for baseline confounding factors between the two groups. All significant variables (set at a *P*‐value < 0.1) were entered in block into the model. A *p* < 0.05 was considered statistically significant. The data coordinating center was established at the Eastern Piedmont University.

#### Ethical issues

The study is a retrospective registry, with anonymized data collection, therefore formal approval from ethical committee was deemed not necessary. However, it was approved by the Ethical Committee of AOU Maggiore della Carità. Novara. The need to notify or ask for approval to the local Ethical Committees was left to each investigator’s discretion according to local and national regulations.

## Results

**Fig. 1:**
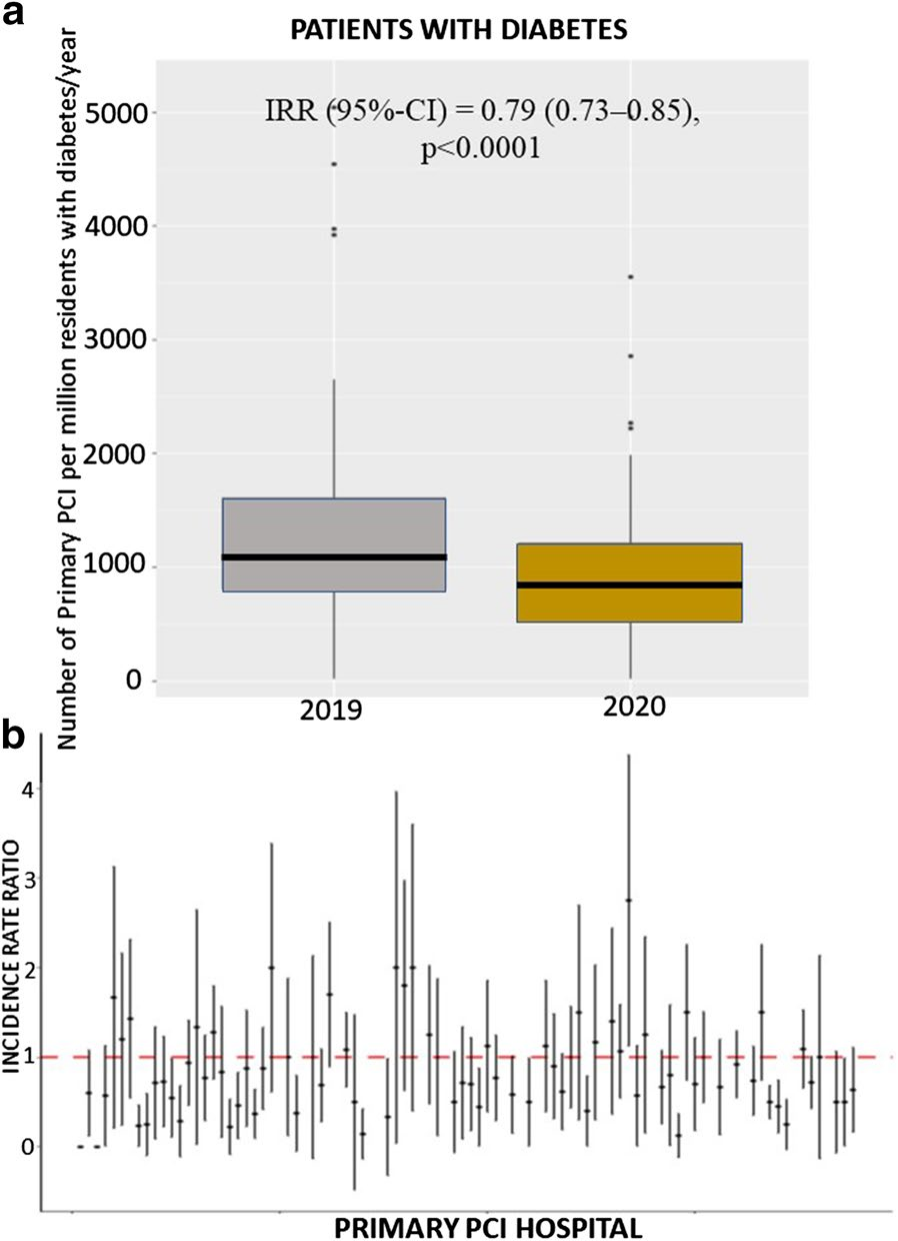
Impact of COVID 19 pandemic on primary PCI cases in patients with diabetes. **a** Box-and-whisker plot showing the number of STEMI patients with diabetes treated by mechanical reperfusion per million of inhabitants with diabetes/year in 2019 and 2020. Whiskers extend to the most extreme data point which is no more than 1.5 times the interquartile range from the box. IRR estimates are based on a Poisson model without covariates. **b** Forest plot of the incidence rate ratio on the log-scaled axis, with 95% confidence interval, across each hospital center

**Fig. 2:**
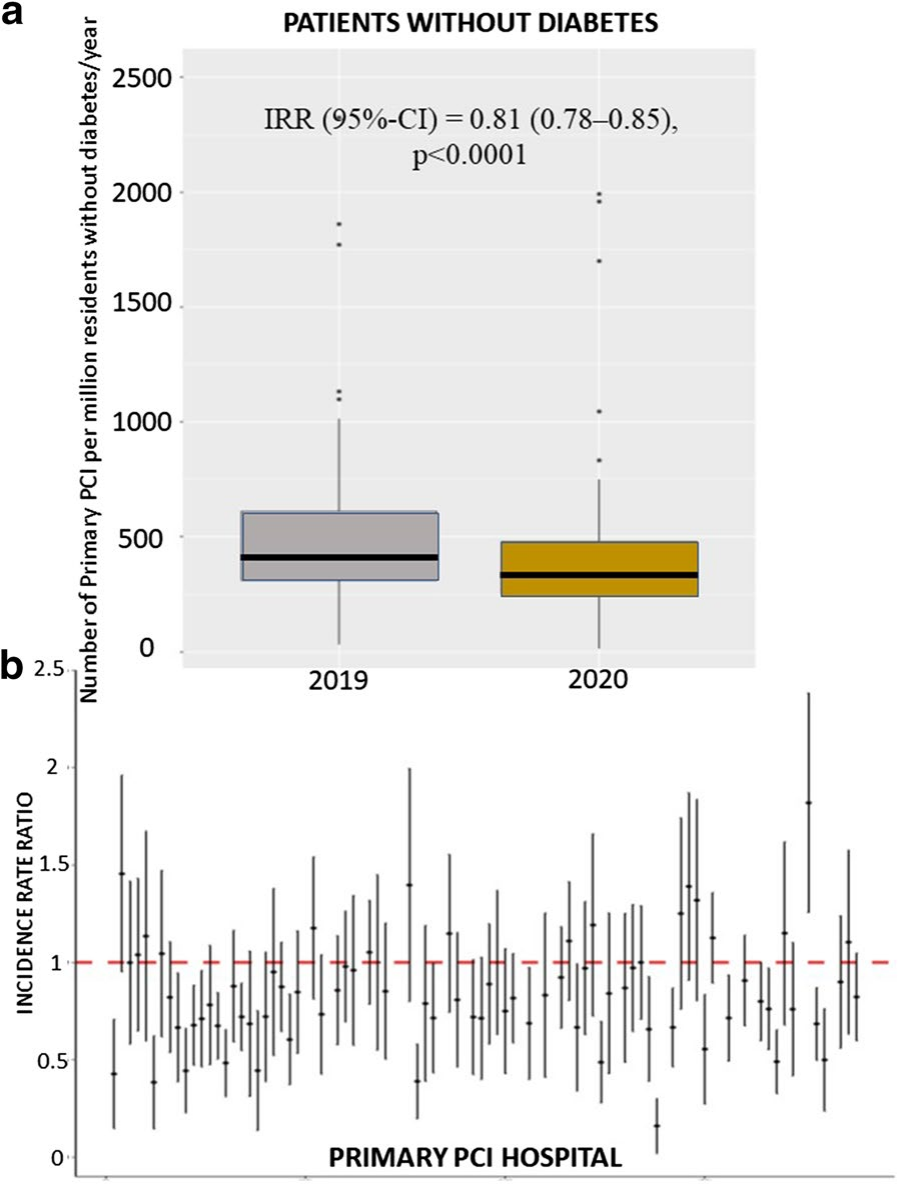
Impact of COVID 19 pandemic on primary PCI cases in patients without diabetes. **a** Box-and-whisker plot showing the number of STEMI patients without diabetes treated by mechanical reperfusion per million of inhabitants without diabetes/year in 2019 and 2020. Whiskers extend to the most extreme data point which is no more than 1.5 times the interquartile range from the box. IRR estimates are based on a Poisson model without covariates. **b** Forest plot of the incidence rate ratio on the log-scaled axis, with 95% confidence interval, across each hospital center

A total of 77 European centers agreed to participate including a total of 6609 STEMI patients undergoing mechanical reperfusion, 3653 patients in 2019 and 2956 patients in 2020. Diabetes was observed in a total of 1356 patients (20.5%), with similar proportions between 2019 and 2020. Among patients with diabetes the number of STEMI treated percutaneously per million residents had a consistent reduction, on average, from 1455 (95%CI 1381 – 1532) in 2019 to 1192 (95% CI 1125- 1262) in 2020 (incidence rate ratio (IRR) 0.79 (95% CI 0.73–0.85), *p* < 0.001) (Fig. 1). A significant heterogeneity was observed among centers (IRR had high variability between centers measured by std error = 0.35, ANOVA Chi-square test for random vs fixed effect Poisson model: *p* < 0.001) (Fig. 1). Similarly, the number of STEMI treated percutaneously per million residents had a consistent reduction, on average, from 518 (95% CI 474–565) in 2019 to 427 (95% CI 387–469) in 2020 in patients without diabetes (IRR was 0.811 (95% CI 0.78–0.84, *p* < 0.0001) (Fig. 2). A significant heterogeneity was observed among centers (IRR had high variability between centers measured by a std error = 0.24, ANOVA Chi-square test for random vs fixed effect Poisson model: *p* < 0.001) (Fig. 2).

**Fig. 3:**
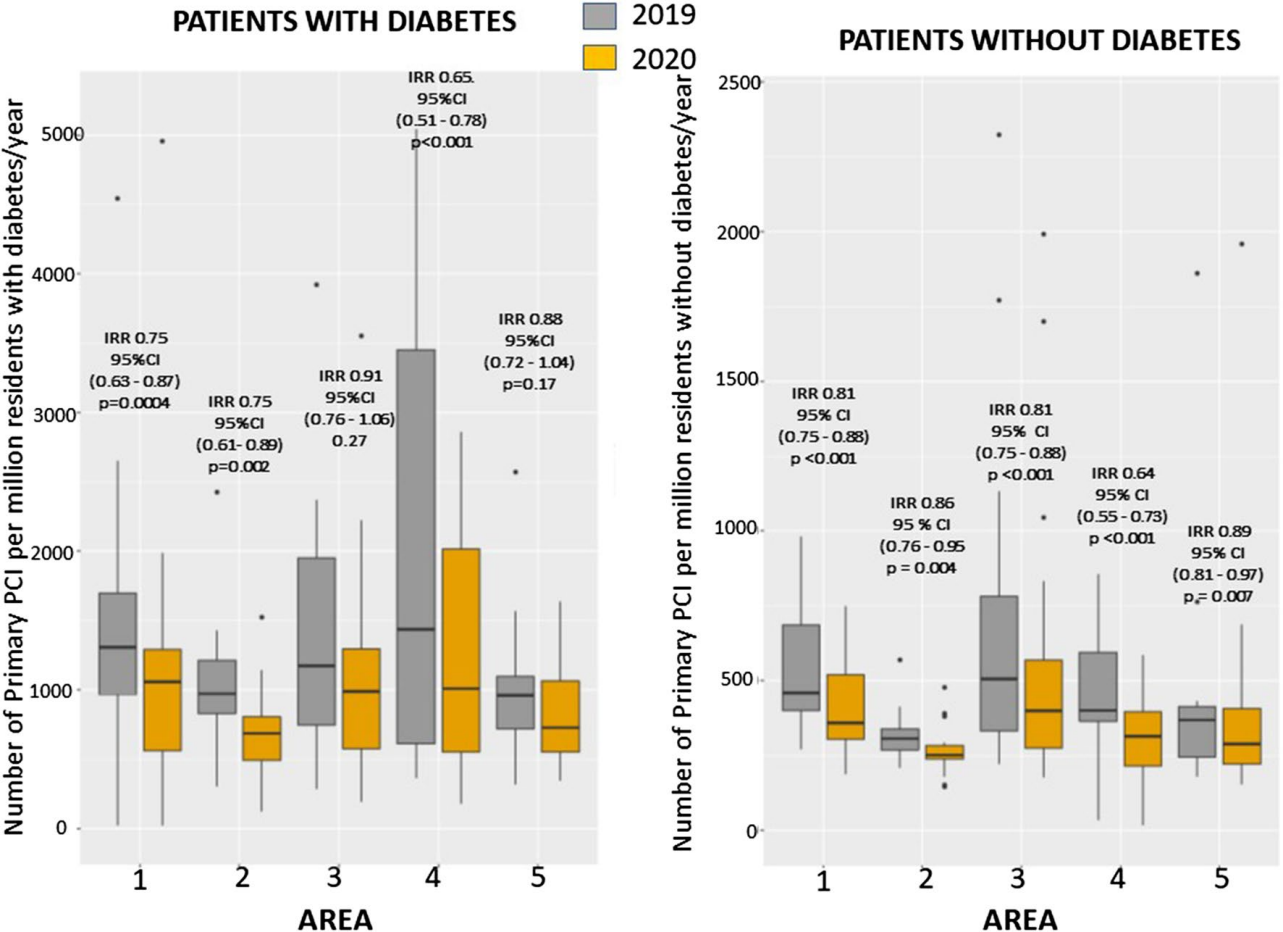
Impact of COVID-19 on PPCI procedures according to geographic area and diabetes. Box-and-whisker plot showing the number of STEMI patients treated by mechanical reperfusion per million of residents/year in 2019 and 2020 across 5 areas in patients with (left graph) and without (right graph) diabetes. A total of 5 European geographical areas were identified: Area 1: Italy; Area 2: Iberian Peninsula (Spain and Portugal); Area 3: Central Europe (France, Germany, The Netherlands, Belgium, Czech Republic); Area 4: Balkan Peninsula (Romania, Slovenia, Greece and North Macedonia); Area 5: North-East Europe (UK, Poland, Finland, Denmark, Russia). Whiskers extend to the most extreme data point which is no more than 1.5 times the interquartile range from the box. IRR estimates are based on a Poisson model without covariates

**Fig. 4:**
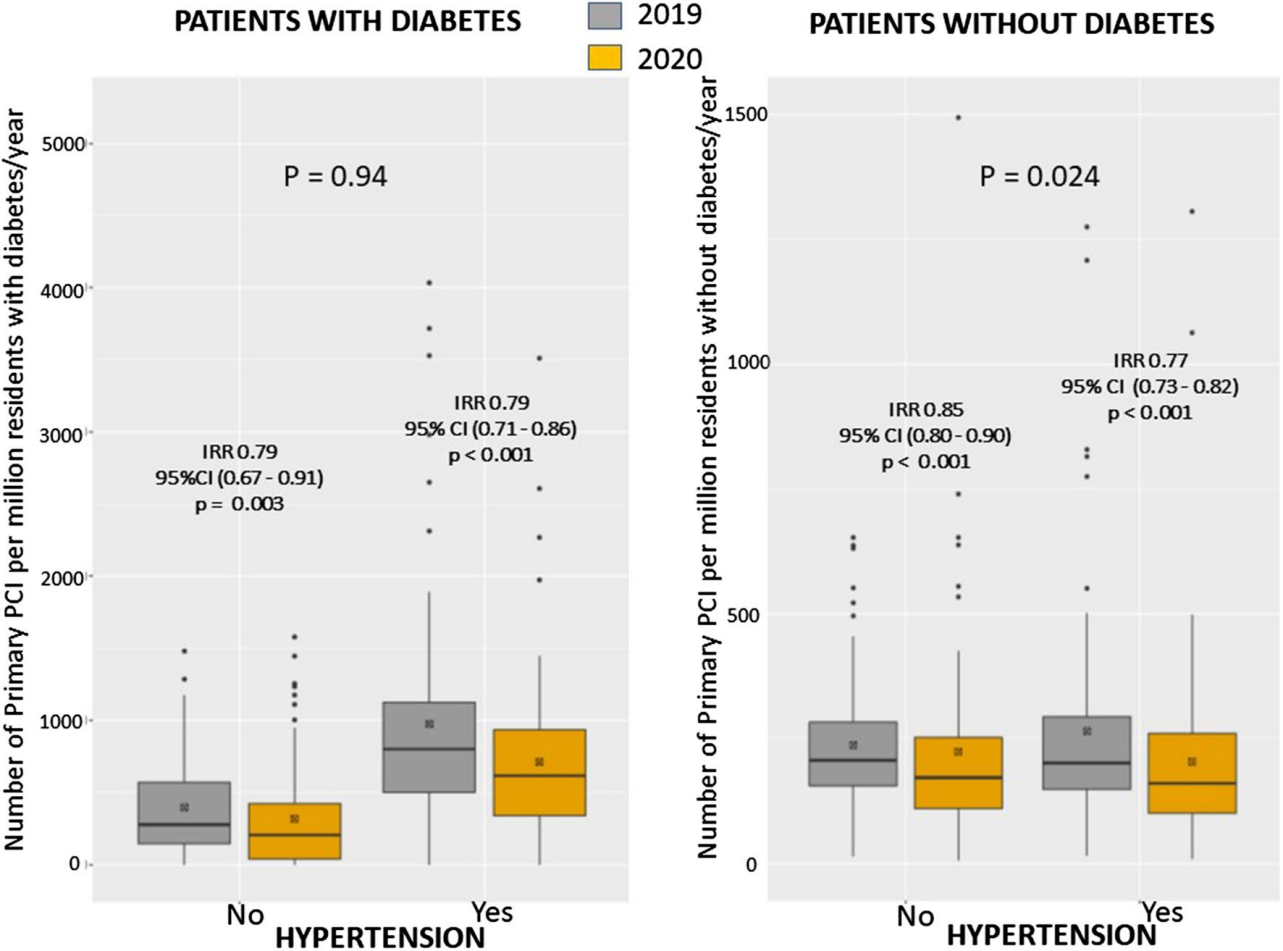
Impact of COVID-19 on PPCI procedures according to Diabetes and Hypertension. Box-and-whisker plot showing the number of STEMI patients treated by mechanical reperfusion per million of residents/year in 2019 and 2020 according to hypertension in patients with (left graph) and without (right graph) diabetes. Whiskers extend to the most extreme data point which is no more than 1.5 times the interquartile range from the box. IRR estimates are based on a Poisson model without covariates. A significant interaction was observed with hypertension

The heterogeneity among centers was not related to the incidence of COVID disease, neither to the COVID-related mortality both at local and national level. (Additional file 1: Figs. S1 and S2). All participating geographic areas had a reduction in STEMI, especially the Balkans (Fig. 3 and Additional file 1: Fig. S3). Furthermore, we used Poisson regression to investigate the reduction in STEMI in both subgroups of subjects with and without diabetes, by age (< = 75, > 75), gender, and hypertension. We found a significant difference in this reduction between patients with (IRR = 0.85 (95% CI 0.80—0.90), p < 0.0001) and without hypertension (IRR 0.78 (95% CI 0.73—0.82) < 0.0001) (Fig. 13S) (p int = 0.024) (Figs. 4 and Additional file 1: Fig. S4). A borderline interaction was observed with gender only in absence of diabetes (p = 0.059) (Additional file 1: Fig. S5). No significant interaction was found for age (Additional file 1: Fig. S6).

### Baseline demographic and clinical characteristics

Individual data analysis was restricted to 6295 patients with complete demographic, clinical procedural and outcome data (complete cases, 95.2%), 3484 in 2019 and 2811 in 2020. Table 1 shows baseline characteristics of patients according to the diabetic status and year of intervention. No difference was observed in baseline characteristics.

**Table 1:** Baseline demographic and clinical characteristics

	No diabetes 2019 (n = 2725)	No diabetes 2020 (n = 2214)	*P* value	Diabetes 2019 (n = 759)	Diabetes 2020 (n = 597)	*P* value
Age (median, IQR)*	63 (55–72)	64 (54–72)	0.56	67 (58–75)	67 (59–75)	0.8
Age > 75 year—n. (%)	554 (20.3)	441 (19.9)	0.72	204 (26.9)	157 (26.3)	0.81
Male gender—n. (%)	2028 (74.4)	1687 (76.2)	0.15	539 (71.0)	408 (68.3)	0.29
Medical hystory						
Hypertension- n (%)	1352 (49.6)	1064 (48.1)	0.28	554 (73.0)	437 (73.2)	0.93
Hypercholesterolemia—n (%)	1033 (37.9)	852 (38.5)	0.68	412 (54.3)	343 (57.5)	0.24
Active Smoker—n (%)	1177 (43.2)	950 (42.9)	0.84	261 (34.4)	205(34.4)	0.98
Family History of CAD—n (%)	674(24.7)	549 (24.8)	0.96	163 (21.5)	109 (18.3)	0.14
Previous STEMI- n (%)	227(8.3)	190 (8.6)	0.93	100 (13.2)	82 (13.7)	0.67
Previous PCI—n (%)	273 (10.0)	240 (10.8)	0.56	166 (21.9)	114(19.1)	0.37
Previous CABG—n (%)	32 (1.2)	33 (1.5)	0.33	27 (3.6)	22 (3.7)	0.90
Geographic area			0.06			0.6
Italy—n (%)	764 (28)	627 (28.3)		200 (26.4)	152 (25.5)	
Iberian Peninsula– n (%)	399 (14.6)	333 (15.0)		150 (19.8)	112 (18.8)	
Central Europe– n (%)	760 (27.9)	609 (27.5)		150 (19.8)	133 (22.3)	
Balkans– n (%)	323 (11.9)	211 (9.5)		131 (17.3)	90 (15.1)	
North-East Europe—n (%)	479 (17.6)	434 (19.6)		128 (16.9)	110 (18.4)	
Referral to primary PCI hospital						
Ambulance (from community)—n (%)	1527 (56)	1327 (59.9)	0.006	367 (48.4)	313 (52.4)	0.14
Time delays						
Ischemia time, median [25—75th]*	180 [120–300]	195 [125–340]	0.002	200 [120–360]	240 [140–420]	0.004
Total Ischemia time < 6 h—n (%) 6–12 h—n (%) 12–24 h—n (%) > 24 h—n (%)	2164 (79.4) 337(12.4) 139 (5.1) 85 (3.1)	1686 (76.2) 291 (13.1) 142 (6.4) 95 (4.3)	0.014	566 (74.6) 100 (13.2) 59 (7.8) 34 (4.5)	423 (70.9) 83 (13.9) 51 (8.5) 40 (6.7)	0.26
Total Ischemia time > 12 h—n (%)	223 (8.2)	237 (10.7)	0.002	93 (12.3)	91 (15.2)	0.11
Door-to-balloon time, median [25—75th]*	34 [21–60]	36 [24–60]	0.012	35 [23–60]	40 [25–64]	0.086
Door-to-balloon time < 30 min—n (%) 30–60 min– n (%) > 60 min– n (%)	1292 (47.4) 827 (30.3) 606 (22.2)	967 (47.3) 715 (32.3) 532 (24.0)	0.032	348 (45.8) 239 (31.5) 172 (22.7)	243 (40.7) 202 (33.8) 152 (25.5)	0.16
Door-to-balloon time > 30 min– n (%)	1433 (52.6)	1247 (56.3)	0.009	410 (54.1)	354 (59.4)	0.051
Clinical presentation						
Anterior STEMI—n (%)	1247 (45.8)	998 (45.1)	0.63	345 (45.5)	281(47.1)	0.55
Out-of-hospital cardiac arrest—n (%)	188 (6.9)	169 (7.6)	0.32	43 (5.7)	29 (4.9)	0.51
Cardiogenic shock—n (%)	193 (7.1)	174 (7.9)	0.30	74 (9.7)	73 (12.2)	0.14
Rescue PCI for failed thrombolysis—n (%)	104 (3.8)	73 (3.3)	0.33	20 (2.6)	22(3.7)	0.27

^*^Mann–Whitney test

*CAD* Coronary Artery Disease, *STEMI* ST-segment Elevation Myocardial Infarction, *PCI* Percutaneous Coronary Intervention, *CABG 0* Coronary Artery Bypass Graft

**Fig. 5:**
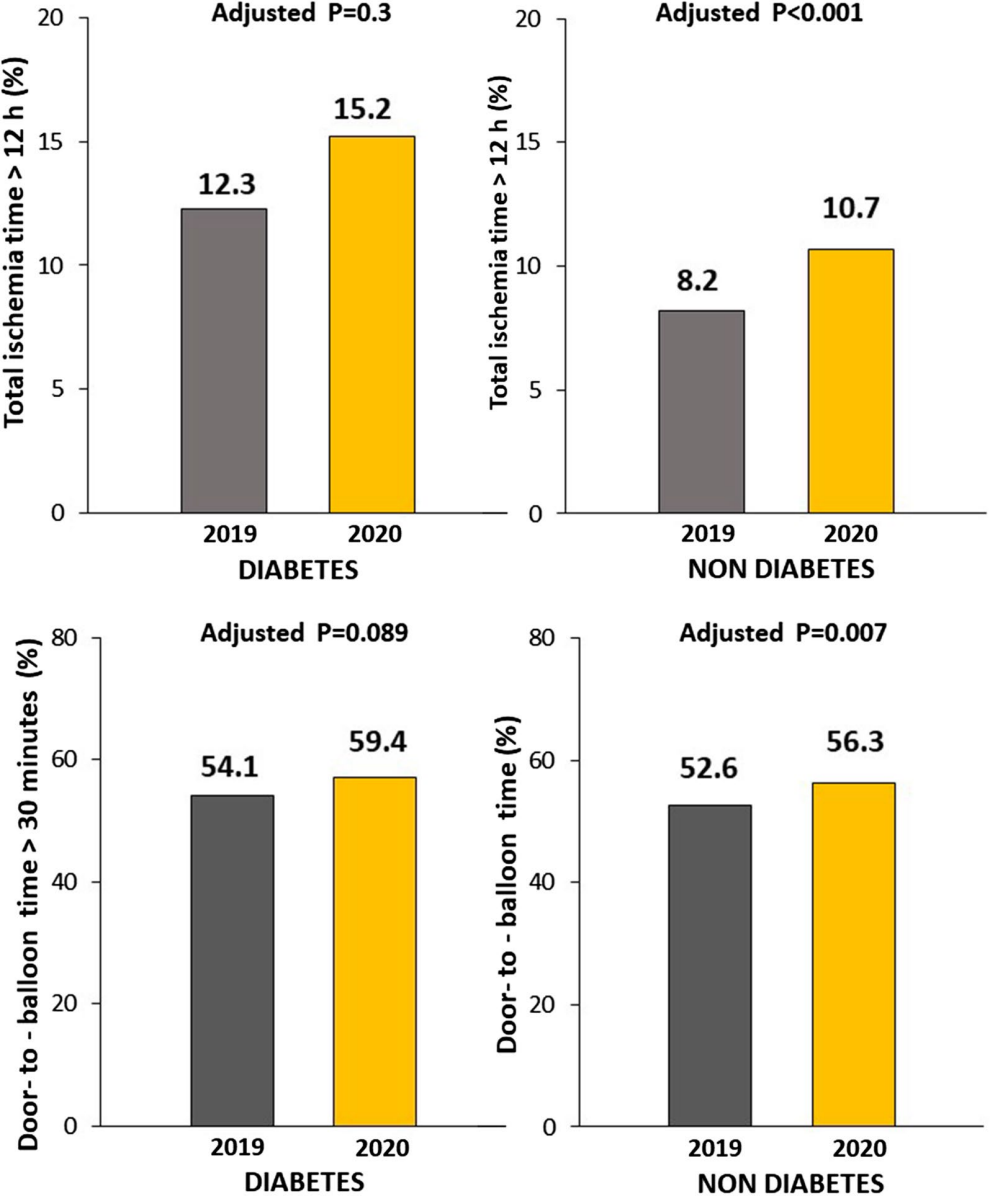
Impact of COVID-19 pandemic on time delays in according to diabetes. Bar Graphs show the association between the year of intervention with time delays (Ischemia time longer than 12 h, upper graphs; Door-to-balloon time longer that 30 min, lower graphs) in both patients with (left graphs) and without (right graphs) diabetes

As shown in Table 1, COVID-19 pandemic was associated with a longer ischemia time, longer DTB time in both patients with and without diabetes (Fig. 5), but a significantly larger use of ambulance only in non diabetic patients. As compared to 2019, radial access and DES were more often used during the pandemic, especially in patients without diabetes, whereas a larger prevalence of multivessel disease during the pandemic was observed only in the diabetic subset.

The association between COVID pandemic and ischemia time longer than 12 h was confirmed after correction for baseline clinical confounders (geographic area, direct access by ambulance, door-to-balloon, radial access, use of DES) in patients without diabetes [Adjusted OR = 1.40 (1.15–1.71), *p* < 0.001], but not among those with diabetes [adjustment for ischemia time, door-to-balloon, multivessel disease, DAPT, use of DES; Adjusted OR = 1.18 (0.86–1.63), *p* = 0.3]. No significant interaction was observed for major risk factors among patients without diabetes (age, p = 0.5; gender, p = 0.32; hypertension, p = 0.49), and with diabetes (age, *p* = 0.07; gender, *p* = 0.59; hypertension, *p* = 0.1). The association between COVID pandemic and door-to-balloon time longer than 30 min was confirmed after correction for baseline clinical confounders (geographic area, direct access by ambulance, door-to-balloon, radial access, use of DES) [Adjusted OR = 1.17 (1.04–1.31), *p* = 0.007] in patients without but not with diabetes [adjustment for ischemia time, door-to-balloon, multivessel disease, DAPT, use of DES; Adjusted OR = 1.21 (0.97–1.51), *p* = 0.089] No significant interaction was observed for major risk factors (no diabetes: age, p = 0.53; gender, p = 0.81; hypertension, *p* = 0.18; diabetes: age, *p* = 0.085; gender, *p* = 0.06; hypertension, p = 0.07).

No difference was observed in the rate of cardiogenic shock at presentation, infarct location, out of hospital cardiac arrest, or rescue procedures after failed thrombolysis.

### Procedural characteristics

Concerning procedural characteristics (Table 2) the use of DES and radial access were more frequent in 2020 (92.7 vs 90.6%, *p* = 0.003) among patients without diabetes, whereas pandemic was associated with a more extensive disease in patient with diabetes (60 vs 54.2%, *p* = 0.032).

**Table 2:** Angiographic and procedural characteristics

	No diabetes 2019 (n = 2867)	No diabetes 2020 (n = 2334)	*P* value	Diabetes 2019 (n = 786)	Diabetes 2020 (n = 622)	*P *value
Radial Access (%)	2316 (85)	1933 (87.3)	0.019	629 (82.9)	486 (81.4)	*0.84*
Culpirt vessel Left main—n (%) Left Anterior Descending Artery—n (%) Circumflex—n (%) Right Coronary Artery—n (%) Anterolateral Branch—n (%) SVG—n (%)	50 (1.8) 1250 (45.9) 386 (14.2) 1021 (37.5) 9 (0.3) 9 (0.3)	35 (1.6) 993 (45) 347 (15.7) 820 (37) 4 (0.1) 12 (0.5)	0.54	20 (2.6) 340 (44.8) 98 (12.9) 291 (38.3) 2 (0.3) 8 (1.1)	17 (2.8) 268 (44.9) 91 (15.2) 209 (35) 2 (0.3) 10 (1.7)	0.64
Proximal Lesion location—n (%)	1343 (49.3)	1115 (50.4)	0.48	389 (51.3)	284 (47.6)	0.43
In-stent Thrombosis—n (%)	97 (3.6)	93 (4.2)	0.24	53 (7.0)	35 (5.9)	0.41
Multivesseldisease—n (%)	1183 (43.4)	988(44.6)	0.39	411 (54.2)	358 (60)	0.032
Preprocedural TIMI 0 flow—n (%)	1674 (61.4)	1404 (63.4)	0.15	440 (58.0)	350 (58.6)	0.81
Thrombectomy—n (%)	526 (19.3)	390 (17.6)	0.13	126 (16.6)	99 (16.6)	0.99
Stenting—n (%)	2509 (92.1)	2042 (92.2)	0.84	681 (89.7)	547 (91.6)	0.23
Drug-elutingstent—n (%)	2480 (91) (91.1)	2057 (92.9)	0.015	677 (89.2)	550 (92.1)	0.068
Postprocedural TIMI 3 Flow—n (%)	2523 (92.6)	2038 (91.9)	0.48	689 (90.3)	529 (88.6)	0.19
Gp IIb-IIIa inhibitors/Cangrelor—n (%)	626 (23.0)	538 (24.3)	0.27	150 (19.8)	133 (22.3)	0.26
Bivalirudin—n (%)	16 (0.6)	9 (0.4)	0.37	8 (1.1)	2(0.3)	0.12
Additional PCI During the index procedure—n (%) Staged—n (%)	389 (14.3) 321 (11.8)	334 (15.1) 294 (13.3)	0.16	111 (14.6) 94 (12.4)	108 (18.1) 67 (11.2)	0.21
DAPT therapy—n (%)	2702 (99.2)	2194 (99.1)	0.82	751 (98.9)	583 (97.7)	0.06

*TIMI* Thrombolysis in Myocardial Infarction, *DAPT* Dual Antiplatelet Therapy

### In-hospital clinical outcome

**Fig. 6:**
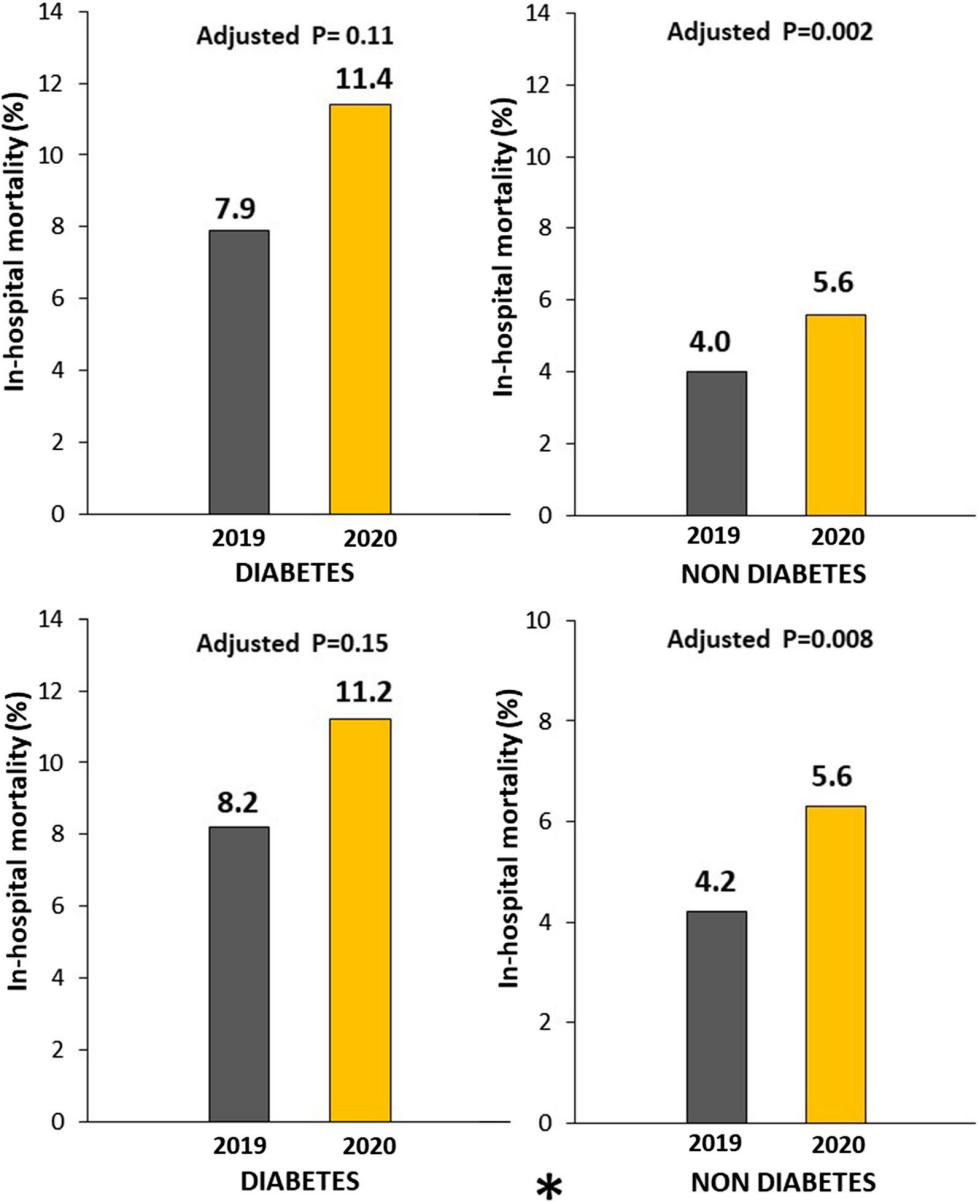
Impact of COVID-19 pandemic on mortality according to diabetes. Bar Graphs show the association between the year of intervention and in-hospital mortality in patients with (upper graphs) and without (lower graphs) COVID positivity. The results are shown in both patients with (left graphs) and without (right graphs) diabetes

A significantly higher mortality was observed in 2020 as compared to 2019 in both patients without ([124, 5.6% vs 109 deaths, 4.0%, OR (95% CI) = 1.42 (1.09–1.85), *p* = 0.009) (Fig. 6) and with diabetes [68 deaths, 11.4% vs 60 deaths, 7.9%, OR (95% CI) = 1.5 (1.04–2.16), *p* = 0.03]. The mortality rate was extremely high among the 62 COVID-19 positive patients in both non diabetic [26.9 vs 4.5%, OR (95% CI) = 7.85 (4.19–14.7), *p* < 0.001] and diabetic group [40 vs 9.2%, OR (95% CI) = 6.57 (1.83–23.6), *p* < 0.001].

The significantly poorer outcomes observed in STEMI patents treated in 2020 persisted after correction for all potential confounding factors (Geographic area, direct access by ambulance, ischemia time, door-to-balloon, radial access, use of DES) in patients without diabetes [Adjusted OR (95% CI) = 1.55 (95% CI 1.18–2.03), *p* = 0.002], but not in patients with diabetes (adjustment for ischemia time, door-to-balloon, multivessel disease, DAPT, use of DES) [Adjusted OR (95% CI) = 1.36 (0.93–1.99), *p* = 0.11].

## Discussion

The ISACS-STEMI COVID 19 registry [12] represents the largest study of STEMI patients undergoing mechanical reperfusion during the COVID pandemic, to date. This subanalysis is the first report investigating the impact of diabetes on STEMI procedures, time delays and outcome based on individual data.

The COVID-19 pandemic has rapidly spread around the world, with 56 million infected and 1.3 million casualties (as of November 18, 2020), especially in Europe, Latin America and United States.

The true impact of COVID-19 on cardiovascular disease and mortality has a long been discussed, with potential direct and indirect effects on occurrence and management of acute heart disease. In has been demonstrated that COVID-19 causes acute cardiac injury that varies from heart failure (HF), myopericarditis to acute MI [15].

Reports about the presence of inflammatory pathophysiological mechanisms, triggering plaque disruption and generating a pro-thrombotic milieu [15–17] supported an anticipated increased in the number of patients presenting with acute coronary syndromes (ACS) during the COVID-19 pandemic. In fact, a recent small report found higher thrombus burden in patients presenting with STEMI and concurrent COVID-19, that certainly contribute to explain the observed poorer outcomes in this population [18].

However, initial small reports from small-sized registries showed a remarkable reduction in the number of patients with ACS. [2–7]. It has been speculated that during the pandemic patients may have avoided acute treatment for fear of COVID infection, or avoidance of burdening an already overwhelmed clinical service. These behaviors may lead to increased morbidity and mortality, especially in STEMI patients in whom a longer time delay has a significant negative impact on myocardial salvage, preservation of left ventricular function, and (short and long-term) survival [19–21].

The ISACS-STEMI COVID-19 [12] is the first largest individual patients’ data based registry that aimed at estimating the true impact of the COVID-19 pandemic on the treatment and outcome of STEMI patients treated by primary percutaneous coronary intervention (PPCI), with identification of ‘at risk’ patient cohorts for failure to present or delays to treatment. This retrospective registry was performed in European high-volume PPCI centers and assessed STEMI patients treated with PPPCI in March/April 2019 and 2020. The global study found that in 2020, during the pandemic, there was a significant reduction in PPCI as compared to 2019 (IRR 811 (95%-CI: 0.78–0.84, p < 0.0001), and mainly for patients with arterial hypertension. Furthermore, the pandemic was associated with a significant increase in door-to-balloon and total ischemia times, which may have contributed to the higher mortality during the pandemic.

However, so far no data have been specifically reported on the impact of diabetes, that was the aim of this subanalysis. In fact, diabetes patients represent an high-risk subgroup of STEMI patients, with described longer delay to treatment [8, 19, 20], and higher thrombotic profile [11] and mortality [8–11], also explained by the presence of more comorbidities, including hypertension [8–11]. Moreover, high prevalence, severity of disease and mortality during Covid-19 infection has been reported among diabetic patients, thus potentially requiring more aggressive management and more strict glycemic control [21, 22].

We found a significant reduction in the number of STEMI patients undergoing mechanical reperfusion, that was similarly observed in both patients with and without diabetes. We found a significant interaction with the decline in procedures in patients with hypertension only among patients without diabetes. Indeed, despite subsequently been disproven [23], the initial alarming reports about the interplay between COVID-19 and the use of ACE-inhibitors and angiotensin receptor blockers (ARBs), that could have increased the expression of ACE2 and patient susceptibility to COVID-19, may have impacted more relevantly in terms of fear of contagion in this group of patients. Moreover, hypertension was among the most common comorbidities declared among patients affected by COVID 19, being associated with worse outcomes, even above other risk factors. In fact, Maddaloni et al. reported that patients with diabetes hospitalized for Covid-19 were at increased risk of adverse outcomes, in case of clustering with cardiometabolic conditions, and especially for hypertension, while patients with a single cardiometabolic risk factor did not differ from patients with no cardiometabolic risk factors [24].

Moreover, hypertension has been associated with vitamin D deficiency, a condition potentially linked to a higher pro-inflammatory status and impaired immune response, that has been suggested to worsen the outcomes in patients with COVID [25].

In addition, we may postulate that the observation of the impact of hypertension only among patients without diabetes, despite its larger prevalence (more than 70%) in diabetic patients, was probably a consequence of the awareness of high cardiovascular risk profile and risk of infarction, as much as the worse clinical presentation of the latter subset of patients, that could have led more often to hospitalization. However, opposite findings were observed in the British Cardiovascular Intervention Society PCI Database Cohort, reporting changes in the characteristics of patients undergoing PCI, particularly for non-ST-segment-elevation myocardial infarction, towards a lower risk phenotype reflecting a more conservative approach among patients with diabetes, hypertension or established coronary artery disease [26].

A longer ischemia time and door-to-balloon time in 2020 as compared to 2019 was observed in both populations. However, after adjustment for all the confounders, the association remained significant only among patients without diabetes. A delayed time from symptoms to first medical contact may be a consequence of both direct patient delay or emergency system related delay, as recently described [27, 28]. In fact, we observed in 2020 a longer ischemia time despite a higher proportion of patients in both groups who were transferred by ambulance from the community to PCI hospitals. Indeed, geographical variations were observed in our study and in previous literature, with no impact of the state of emergency due to COVID-19 being observed in certain countries, and increased mortality in other regions [29, 30].

These findings contributed to explain the results in terms of mortality. In fact, patients admitted in 2020 had a significantly higher mortality as compared to those admitted in 2019, in both patients with and without diabetes. However, after adjustment for all confounders, the association remained significant only in patients without diabetes.

Importantly, the COVID positive population represented a very high-risk subgroup, in both groups of patients with and without diabetes, confirming previous reports [7, 31].

Several actions should be attempted by scientific societies and health authorities in order to highlight the importance of recognition and response to characteristic symptoms of acute myocardial infarction, especially among patients suffering from hypertension. In fact, recent studies in different populations and with different designs arrived at the consistent message that the continued use of ACE-inhibitors and ARBs is unlikely to be harmful in patients with COVID-19 and this may certainly reduce any fear of contagious for these patients [32–34]. In particular, in the recent randomized BRACE-CORONA Trial [35], 659 patients with chronic RASI therapy at admission and confirmed diagnosis of COVID-19 were randomly assigned to a temporary 30-day suspension or continuation of RASI therapy. A similar 30 day mortality (2.8% vs 2.7%) was observed between the two groups.

## Limitations

This study is limited by its retrospective design. It was conducted during a pandemic emergency, which was challenging and expected to encounter missing data. data. Nevertheless, our main data analysis and conclusions are based on counts and, therefore, the overall cohort of patients was included. Furthermore, even in the analysis based on full individual patients data, this limitation and the potential risk of type II error was largely overcome by the high complete case series (> 95%) and the high statistical power due to the size of the study population. We did routinely collect information on chronic therapies at admission. However, based on the larger prevalence of cardiovascular risk factors, we may expect a larger use of antihypertensive, statin and antiplatelet therapies in patients with diabetes, that could have provided potential protective effects.

Furthermore, our study was conducted mainly in European countries, therefore limiting the applicability of our results to other regions with younger populations and limited healthcare resources. Finally, even though we did not find any difference in out-of-hospital cardiac arrest, we cannot exclude that the reduction in STEMI patients observed in 2020 may have resulted from higher rates of prehospital death due to longer delays to first medical contact, as has been described during the COVID-19 pandemic [25, 26].

## Conclusions

The COVID-19 pandemic had a significant impact on the treatment of patients with STEMI, resulting in a reduction in primary PCI procedures, especially among patients without diabetes suffering from hypertension, and in a longer delay to treatment, which may have contributed to the increased mortality during this pandemic, especially in this subset of patients. Our data suggest that health authorities, supported by scientific societies, should take vigorous action to prevent patients from neglecting characteristic symptoms of an acute myocardial infarction, especially among patients who suffer from hypertension.

## Supplementary Information

**Additional file 1.** Supplementary figures.

## Data Availability

Upon request to investigators.

## Abbreviations

COVID-19: Coronavirus disease 2019
STEMI: ST- segment elevation myocardial infarction
ACS: Acute coronary syndrome
PCI: Percutaneous coronary intervention
IRR: Incidence rate ratio
OR: Odds ratio
