# Supplementary material for: Impact of COVID-19 pandemic and diabetes on mechanical reperfusion in patients with STEMI: insights from the ISACS STEMI COVID 19 Registry
Source: Cardiovasc Diabetol. 2020 Dec 18;19:215. doi: 10.1186/s12933-020-01196-0 (PMC7747477; doi:10.1186/s12933-020-01196-0)
Supplement: Supplementary file 1 — Additional file 1. Supplementary figures. [file 12933_2020_1196_MOESM1_ESM.pptx]

## Slide 1
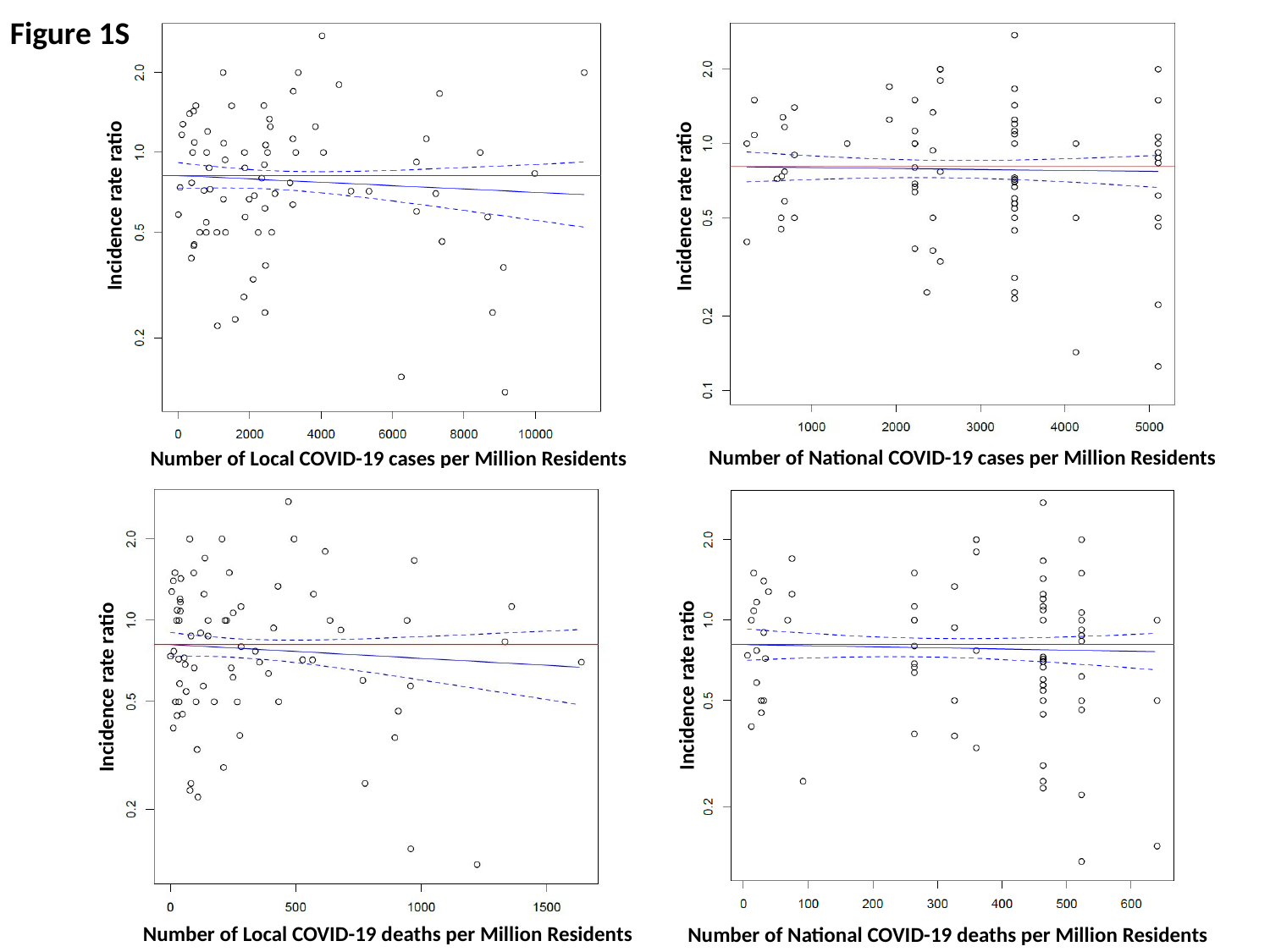

Figure 1S
Incidence rate ratio
Incidence rate ratio
Number of National COVID-19 cases per Million Residents
Number of Local COVID-19 cases per Million Residents
Incidence rate ratio
Incidence rate ratio
Number of Local COVID-19 deaths per Million Residents
Number of National COVID-19 deaths per Million Residents

## Slide 2
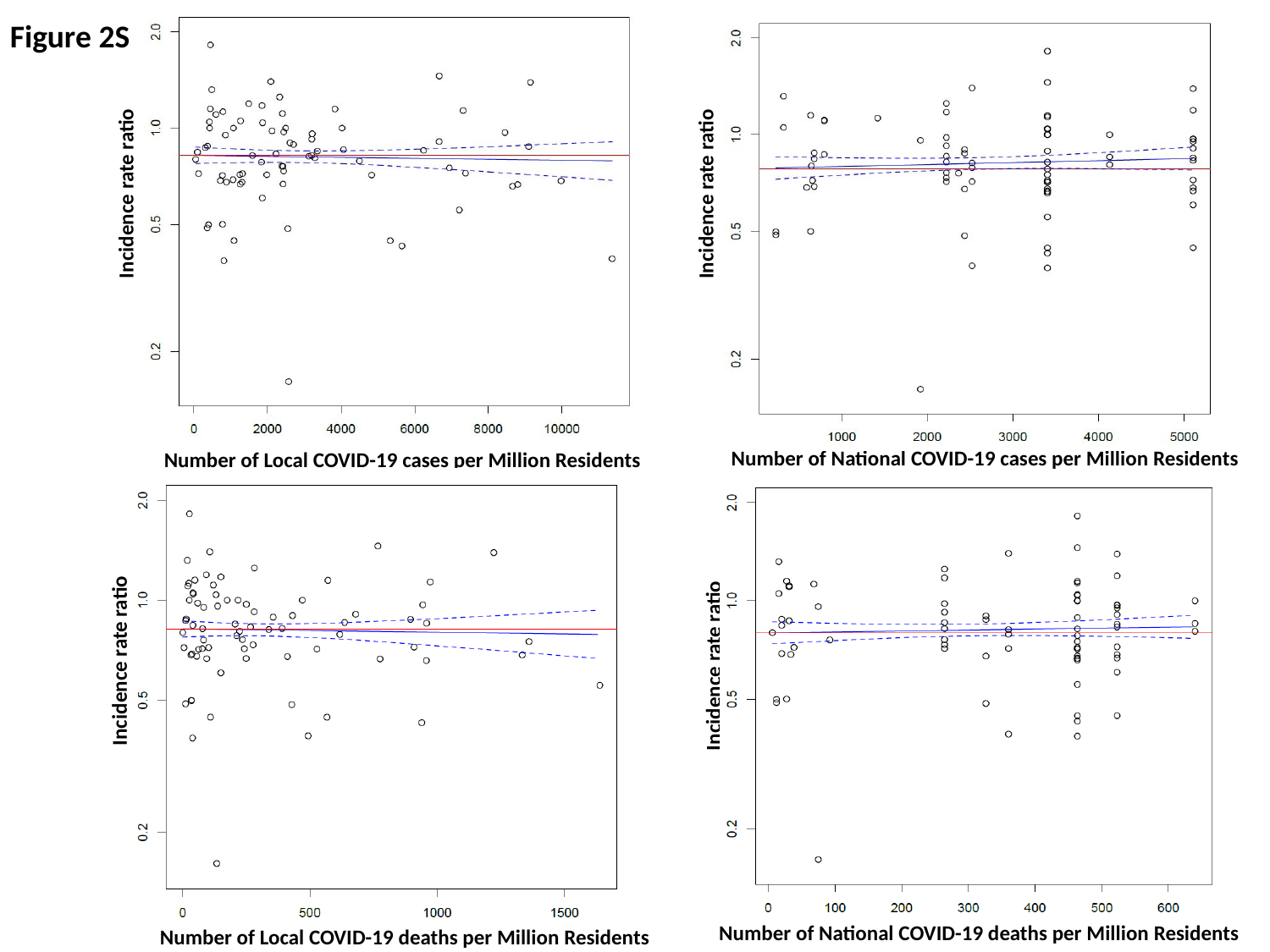

Figure 2S
Incidence rate ratio
Incidence rate ratio
Number of National COVID-19 cases per Million Residents
Number of Local COVID-19 cases per Million Residents
Incidence rate ratio
Incidence rate ratio
Number of National COVID-19 deaths per Million Residents
Number of Local COVID-19 deaths per Million Residents

## Slide 3
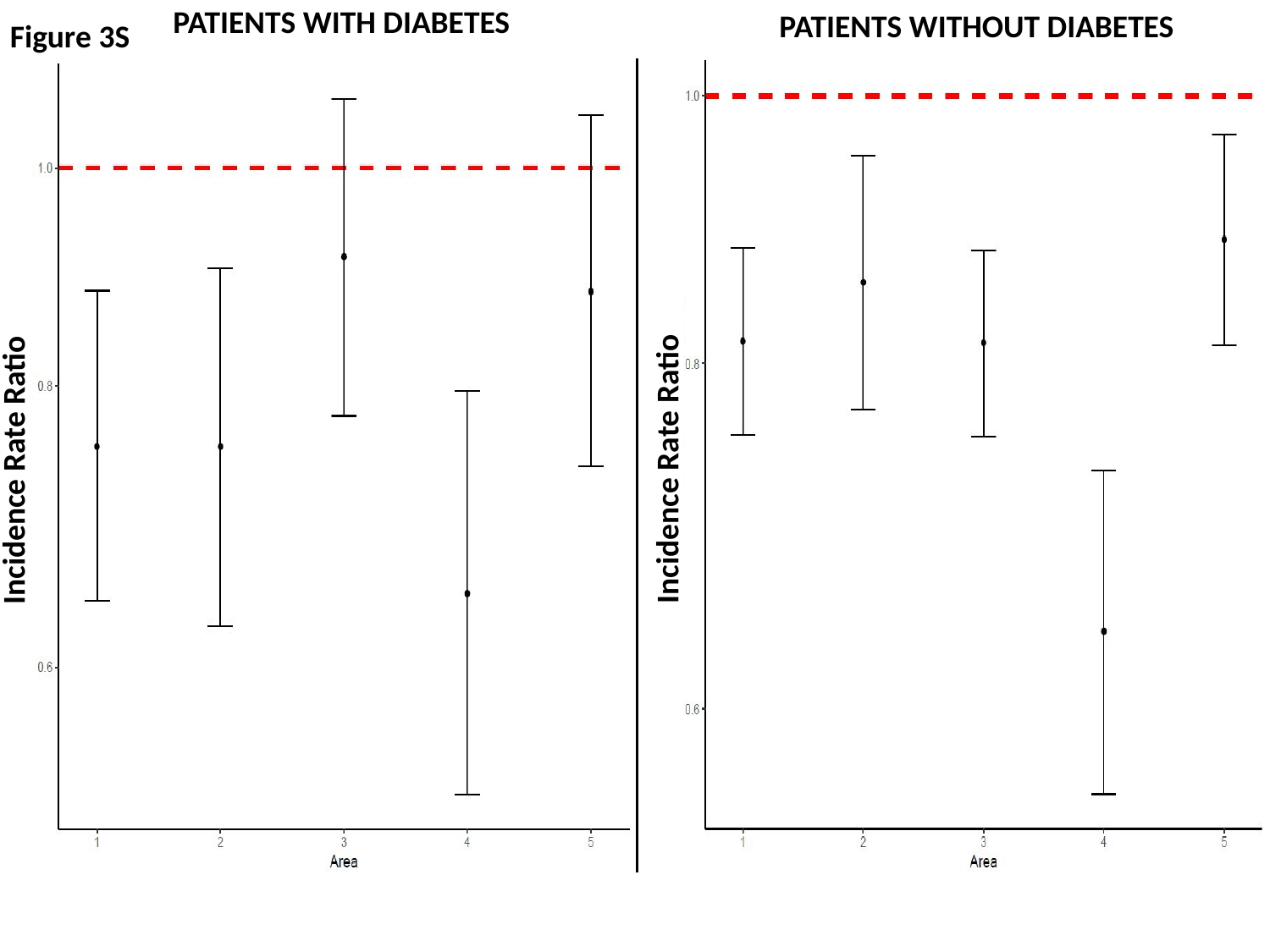

PATIENTS WITHOUT DIABETES
PATIENTS WITH DIABETES
Figure 3S
Incidence Rate Ratio
Incidence Rate Ratio

## Slide 4
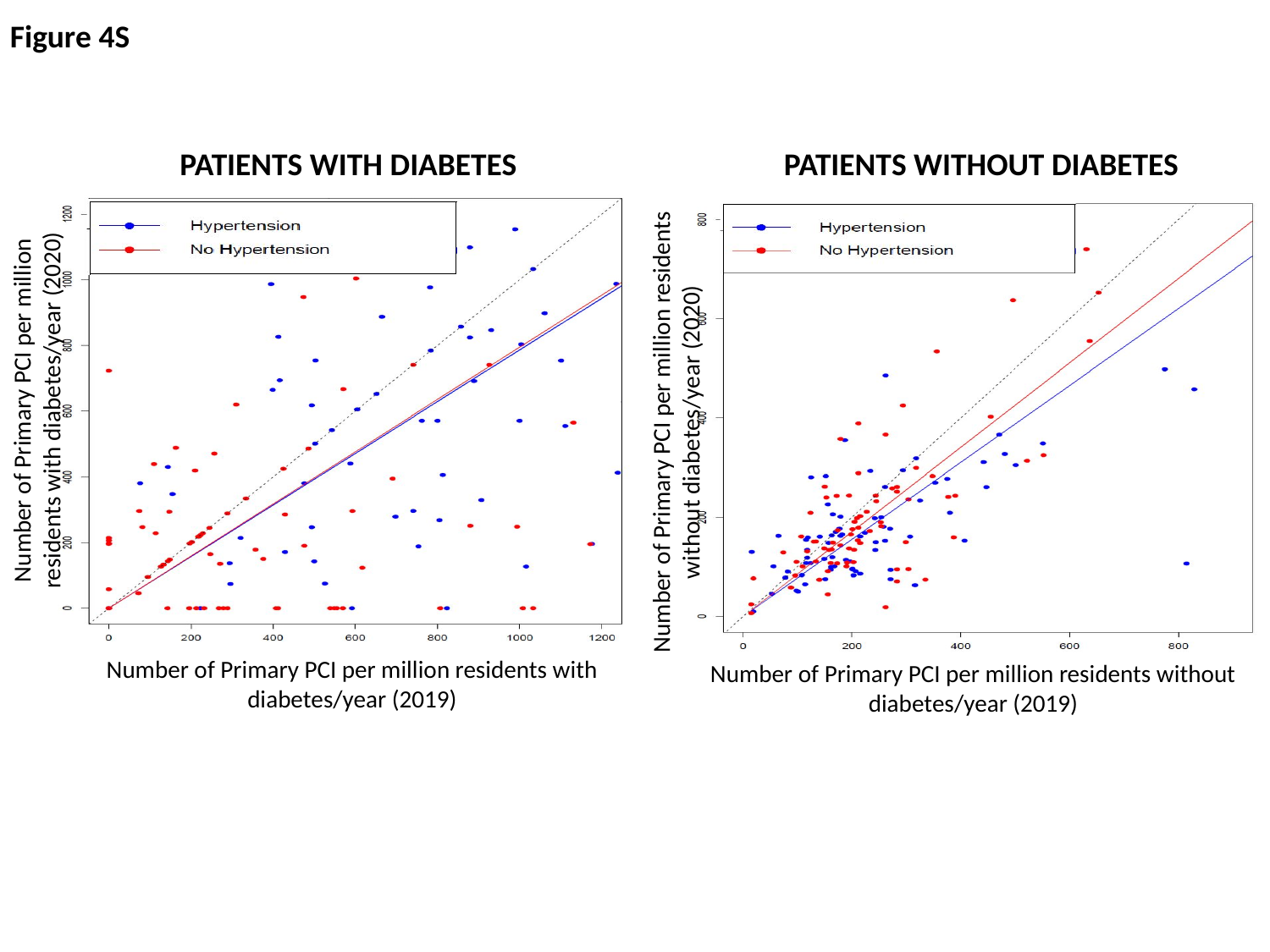

Figure 4S
PATIENTS WITH DIABETES
PATIENTS WITHOUT DIABETES
Number of Primary PCI per million residents with diabetes/year (2020)
Number of Primary PCI per million residents without diabetes/year (2020)
Number of Primary PCI per million residents with diabetes/year (2019)
Number of Primary PCI per million residents without diabetes/year (2019)

## Slide 5
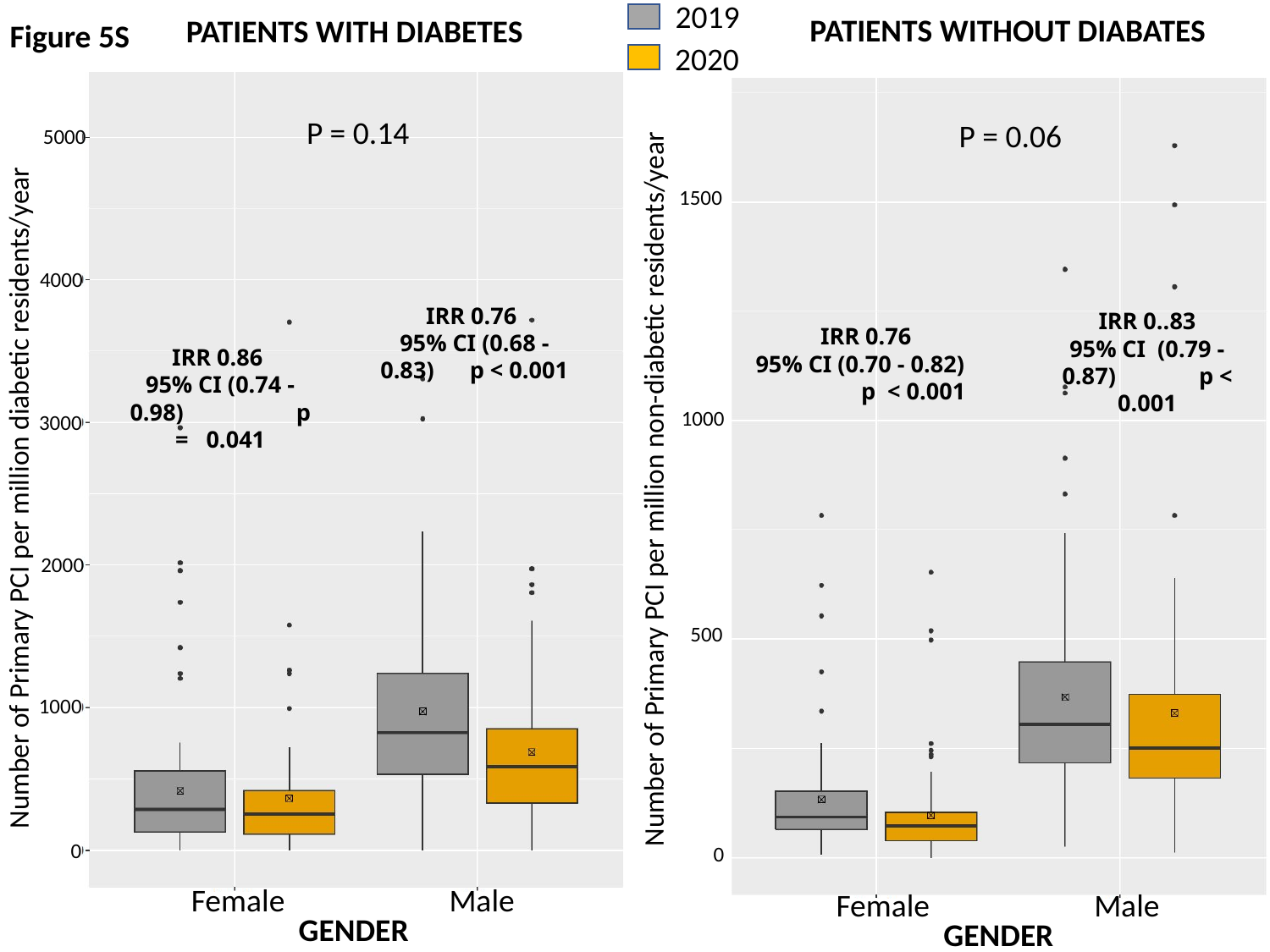

2019
PATIENTS WITHOUT DIABATES
PATIENTS WITH DIABETES
Figure 5S
2020
P = 0.14
P = 0.06
5000
1500
4000
IRR 0.76
95% CI (0.68 - 0.83) p < 0.001
IRR 0..83
95% CI (0.79 - 0.87) p < 0.001
IRR 0.76
95% CI (0.70 - 0.82) p < 0.001
IRR 0.86
95% CI (0.74 - 0.98) p = 0.041
1000
3000
Number of Primary PCI per million diabetic residents/year
Number of Primary PCI per million non-diabetic residents/year
2000
500
1000
0
0
Female
Male
Female
Male
GENDER
GENDER

## Slide 6
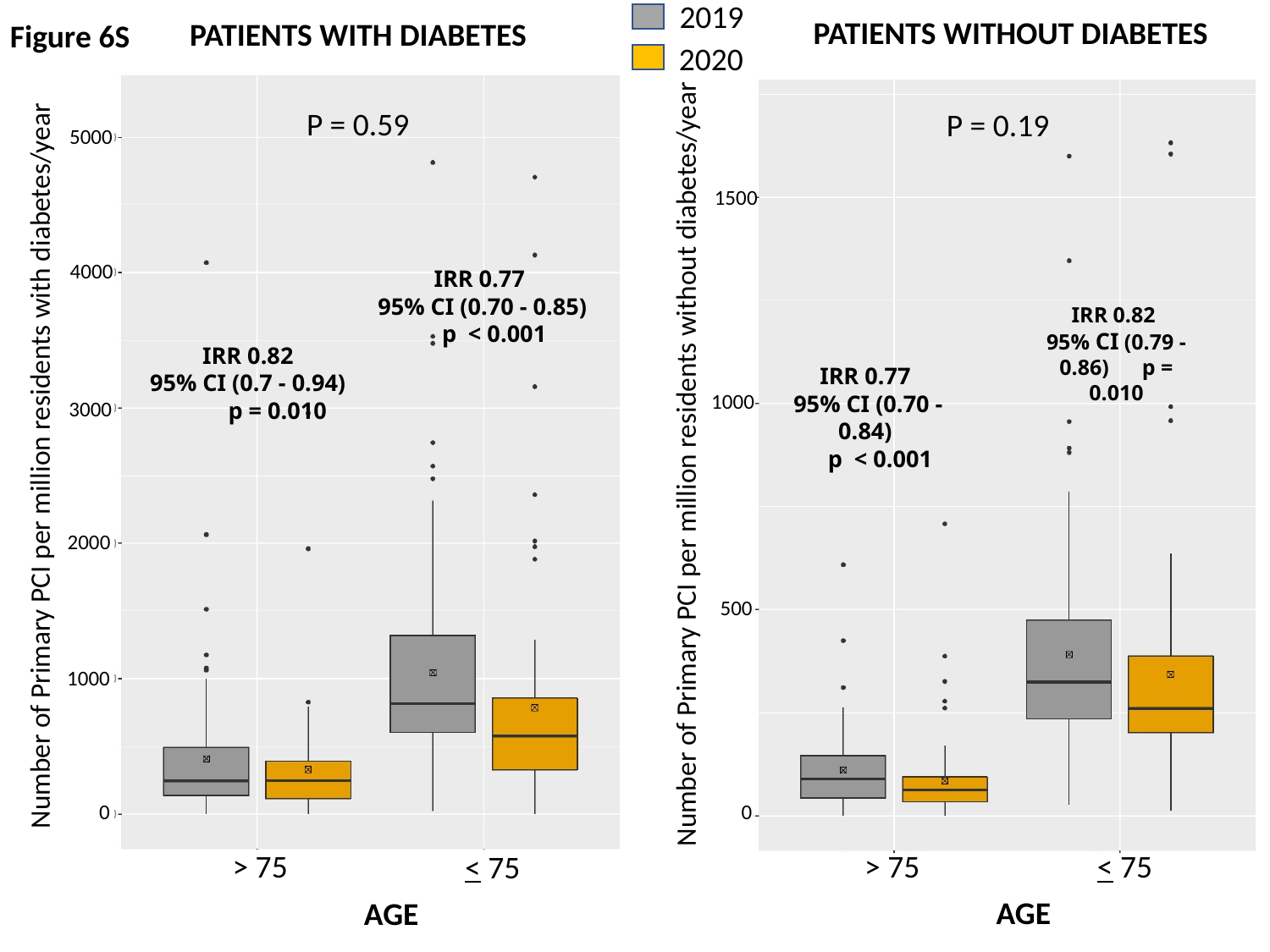

2019
PATIENTS WITHOUT DIABETES
PATIENTS WITH DIABETES
Figure 6S
2020
P = 0.59
P = 0.19
5000
1500
4000
IRR 0.77
95% CI (0.70 - 0.85)
 p < 0.001
IRR 0.82
95% CI (0.79 - 0.86) p = 0.010
IRR 0.82
95% CI (0.7 - 0.94) p = 0.010
IRR 0.77
95% CI (0.70 - 0.84)
 p < 0.001
1000
3000
Number of Primary PCI per million residents with diabetes/year
Number of Primary PCI per million residents without diabetes/year
2000
500
1000
0
0
> 75
> 75
< 75
< 75
AGE
AGE
